# Maternal Anti-Ro/SSA Autoantibodies and Prolonged PR Interval in a Competitive Athlete: Beyond Training-Induced Electrical Remodeling

**DOI:** 10.1016/j.jaccas.2022.05.032

**Published:** 2022-09-07

**Authors:** Chiara Fusi, Pietro Enea Lazzerini, Luna Cavigli, Marta Focardi, Maurizio Acampa, Matteo Cameli, Serafina Valente, Flavio D'Ascenzi

**Affiliations:** a Department of Medical Biotechnologies, Division of Cardiology, University of Siena, Siena, Italy; b Department of Medical Sciences, Surgery and Neurosciences, University of Siena, Italy; c Stroke Unit, University Hospital of Siena, Italy

**Keywords:** autoimmune, bradycardia, electrocardiogram, exercise, immunization, anti-Ro/SSA, anti-Sjögren-syndrome-related antigen A antibodies, AV, atrioventricular, ECG, electrocardiogram

## Abstract

Prolongation of the PR interval is common among competitive athletes. However, further investigations should be performed when the PR interval is markedly prolonged. We report the case of a young male athlete with an autoimmune-mediated atrioventricular block due to circulating anti-Ro/SSA-antibodies in the mother (late progressive congenital form). (**Level of Difficulty: Advanced.**)

## Central Illustration

**Figure:**
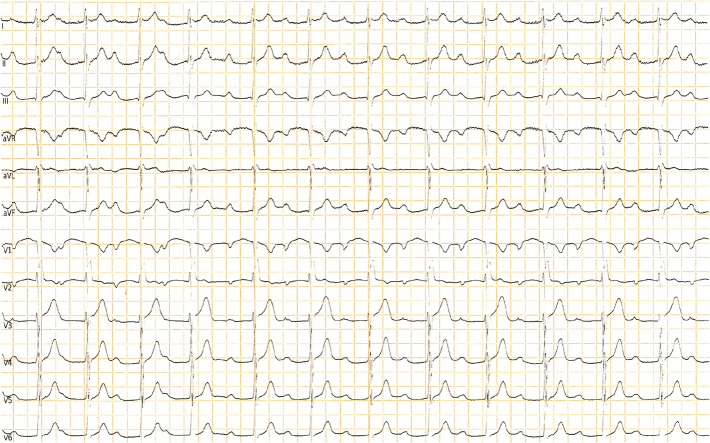

**Figure 1:**
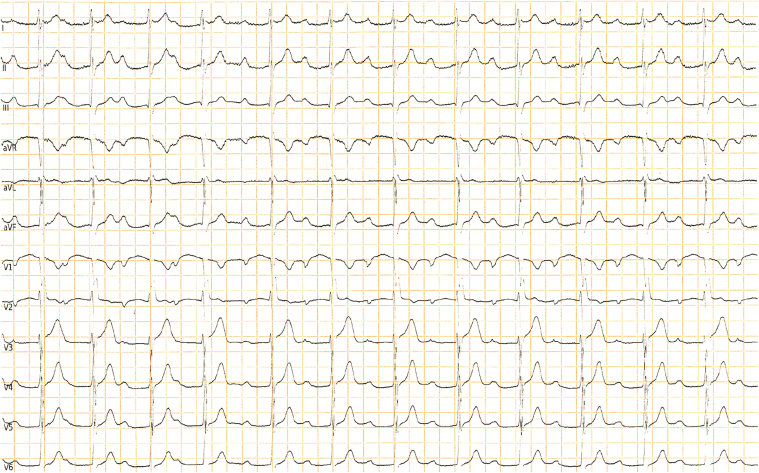
12-Lead Resting Electrocardiogram at Presentation Resting heart rate of 75 beats/min, PR interval of 400 ms, and incomplete right bundle branch block.

**Figure 2:**
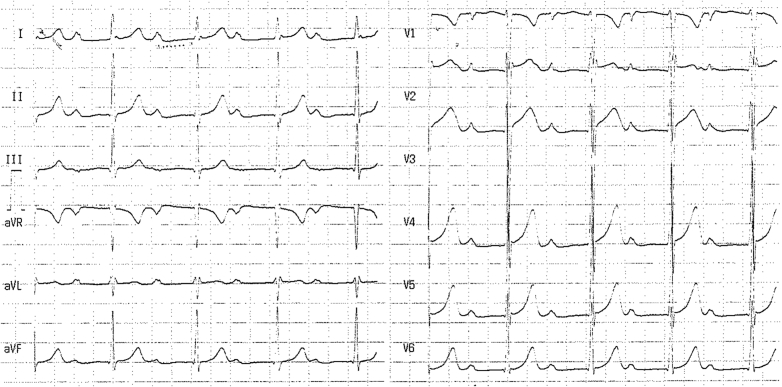
12-Lead Resting Electrocardiogram at Age 10 Years PR interval of 400 ms.

**Figure 3:**
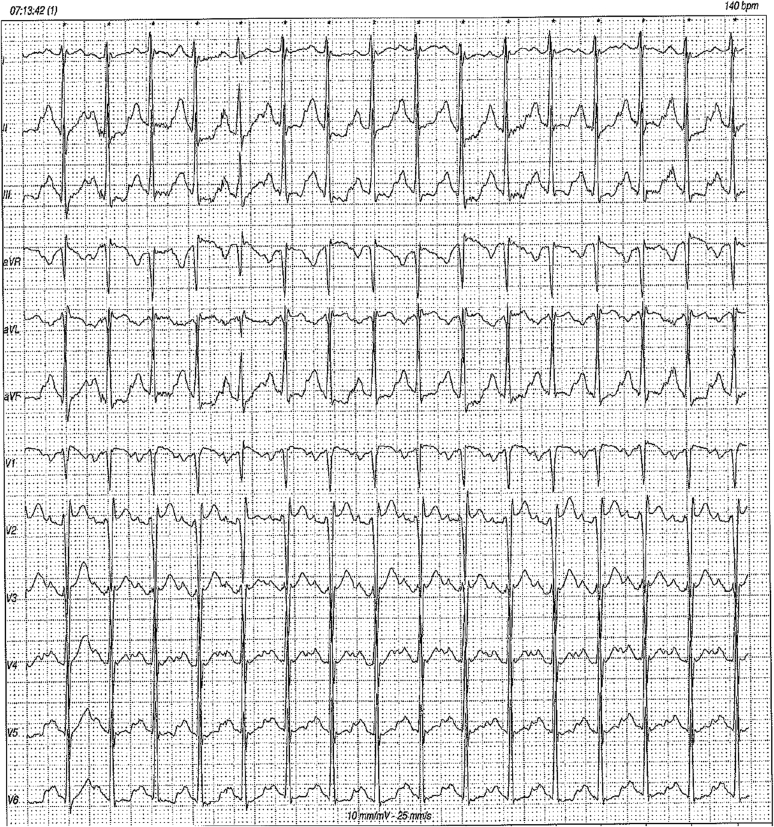
12-Lead 24-Hour Ambulatory Electrocardiogram Monitoring Shortening of PR interval during the training session.

A 26-year-old male athlete was referred to our center because of a significant prolongation of the PR interval (PR = 400 ms) (Figure 1). At the age of 7 years, he underwent a preparticipation screening that revealed for the first time this conduction disturbance (PR 400 ms), which was confirmed when he was 10 years old (Figure 2). Since that age, he had played soccer for 6 h/wk without symptoms or limitations. The yearly cardiovascular screening was completed by ambulatory 12-lead electrocardiogram (ECG) monitoring, including a training session, showing 1° and 2° type 1 atrioventricular (AV) block episodes with rare, intermittent nonconducted P waves during the night and the absence of pauses. A shortening of the PR interval was observed during the training session, with a partial adaptation during the effort (Figure 3). His blood pressure was 130/80 mm Hg, resting heart rate 75 beats/min, respiratory rate 16 breaths/min, and oxygen saturation 99% on room air during the first evaluation. The result of his physical examination was normal. His blood test results were normal, with negative inflammation indices and normal thyroid function.

**Learning Objectives**

• To evaluate whether low-grade atrioventricular block in competitive athletes is a sign of an athlete’s heart or a phenotypic expression of a cardiac disorder.
• To select appropriate diagnostic workup in young athletes with atrioventricular block to give specific indications to sports practice, therapeutic options, and follow-up.
• To consider autoimmune causes of an atrioventricular block presenting in childhood/adulthood and congenital atrioventricular block.

## Medical History

The patient did not have a family history of sudden cardiac death, channelopathies, or cardiomyopathies. His personal remote medical history was unremarkable. Notably, although marked PR prolongation (400 ms) was already present at the age of 7 years, the neonatal ECG documented a normal PR interval. The patient did not have any siblings.

## Differential Diagnosis

According to current recommendations, 1° AV block (up to 200-399 ms) and 2° type 1 AV block can be considered physiological adaptations to exercise training and do not require further testing, particularly if they disappear during exercise and detraining.^1^ Otherwise, further investigations are recommended to exclude abnormalities associated with sudden cardiac death in athletes, such as -structural cardiomyopathies, channelopathies, and autoimmunity, and to guide indications to sports practice, therapeutic options, and follow-up.^1^

Considering that AV blocks in children can be linked to structural heart disease, the physician should exclude the most common congenital or acquired cardiac diseases: L-transposition of the great arteries and inflammatory or infiltrative processes.^2^ Then, it is necessary to rule out primary electrical disease linked to variants in ion channel genes (SCN5A, SCN1B, SCN10A, TRPM4, KCNK17) and cardiac connexin proteins genes.^3^^,^^4^ Finally, 1 possible cause of childhood AV blocks is congenital or acquired autoimmunity, and differential diagnosis is fundamental as it carries prognostic and therapeutic implications.^5^^,^^6^ Table 1 summarizes the main congenital and acquired causes of AV block in the young. Table 2 summarizes the main clinical characteristics of autoimmune (anti-Ro/SSA-associated) forms of AV blocks.

**Table 1:** Congenital and Acquired Causes of Atrioventricular Block in the Young

Congenital	Acquired
Autoimmunity (anti-Ro/SSA-associated): congenital, late progressive congenital Structural heart disease: L-transposition of great arteries, left atrial isomerism, atrial septal defects, endocardial cushion defects Primary electrical disease: variants in ion channel genes, cardiac connexin proteins genes	Inflammatory: myocarditis, endocarditis, acute rheumatic fever, Lyme carditis, systemic lupus erythematosus and other CTDs, spondyloarthritis Infiltrative: amyloidosis, sarcoidosis, scleroderma Postoperative: atrial surgery Other: autoimmune (anti-Ro/SSA-associated), drugs/toxins, electrolyte disturbances, Chagas disease, hypothyroidism, neuromuscular dystrophies, high levels of vagal tone

Anti-Ro/SSA = anti-Sjögren-syndrome-related antigen A antibodies; CTDs = connective tissue diseases.

**Table 2:** Autoimmune (Anti-Ro/SSA-Associated) atrioventricular Blocks

Clinical Characteristic	Congenital	Late Progressive Congenital	Acquired
Mother/child immunologic findings	Circulating anti-Ro/SSA in the mother and in the patient	Circulating anti-Ro/SSA in the mother (not in the patient)	Circulating anti-Ro/SSA in the patient (not in the mother)
Incidence	1 in 15,000-25,000 live births	Not well known (possibly at least 10% of all cases of isolated 3° AVB of unknown origin in adults)	Not well known (possibly at least 10% of all cases of isolated 3° AVB of unknown origin in adults)
Age at onset	In utero/at birth/within the neonatal period (0-27 days)	Childhood/adulthood	Adulthood
Presentation	Progressively developing conduction disturbances (early phase: reversible 1° and 2° AVB; final phase: irreversible congenital 3°AVB)	Subclinical AV node damage during the fetal/neonatal period; late first diagnosis of AVB during childhood or adult age	Unexplained (idiopathic) AVB in the adult age
Clinical concerns	15%-30% mortality rate, mostly in utero/early postnatal life	Worsening of AVB with age (accelerated conduction system senescence)	Pacemaker implantation may be avoided or delayed
Response to therapy	Complete AVB in the fetus is almost always irreversible; IVIG and fluorinated steroids can prevent progression to 3°AVB	Immunosuppressive treatment is ineffective.	Responsive to immunosuppressive therapy

anti-Ro/SSA = anti-Sjögren-syndrome-related antigen A antibodies; AV = atrioventricular; AVB = atrioventricular block; IVIG = intravenous immunoglobulins.

## Investigations

**Figure 4:**
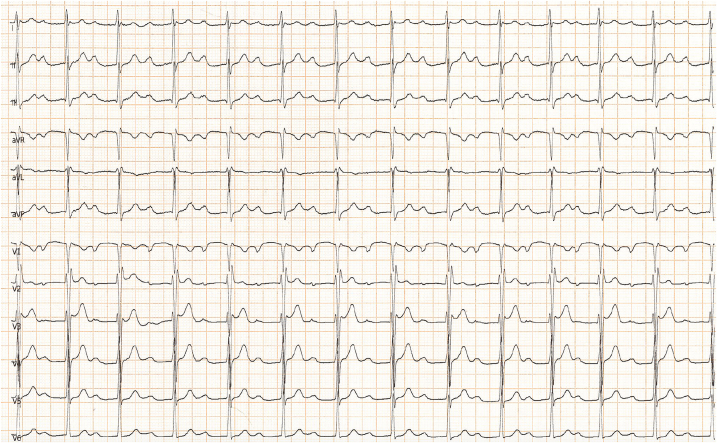
12-Lead Resting Electrocardiogram After 3 Months of Detraining No significant variation in PR interval.

The 12-lead resting ECG showed a heart rate of 75 beats/min, PR interval of 400 ms, and incomplete right bundle branch block. The athlete underwent 12-lead 24-hour ambulatory ECG monitoring, including a training session, to assess the chronotropic response to exercise: we demonstrated a 1° and 2° type 1 AV block with narrow QRS without pauses and a shortening of the PR interval during the training session, also confirmed by exercise testing. Given that the autonomic nervous system regulates the AV node refractory period, the PR interval is expected to decrease during exercise together with changes in sympathetic activity. Otherwise, the worsening of conduction during the effort would suggest a disease of the His-Purkinje system (intranodal block). Nevertheless, marked prolongation of the PR interval in the absence of bradycardia in a nonprofessional athlete suggested the possibility that exercise was not responsible for this disturbance. To complete the assessment of the potential contribution of training-induced electrical remodeling, we advised 3 months of detraining. Notably, after this period, the 12-lead resting ECG showed no significant variation in PR interval (Figure 4), further supporting the hypothesis that this abnormality was not entirely related to training and parasympathetic hypertonia.

Because the results of standard and advanced echocardiography, cardiac magnetic resonance, blood testing, and the genetic test did not demonstrate any abnormalities, we investigated the autoimmunity of the patient and his mother, given the potential association between damage induced by anti-Ro/SSA and marked prolongation of the PR interval.^5^ His mother did not have any history of the rheumatologic disease; nevertheless, we found maternal seropositivity, suggesting the hypothesis of a late progressive congenital form of autoimmune anti-Ro/SSA-associated AV block^7^ in which the damage to the conduction system of the fetus develops in utero, mediated by the transplacental passage of antibodies produced by the mother and crossreacting with calcium channels. The conduction system injury presents subclinically at birth in these individuals and becomes evident later in life.^7^ Accordingly, no circulating anti-Ro/SSA-autoantibodies were found in the athlete.

## Management and Follow-Up

Given the isolated maternal autoantibody positivity, the patient was not considered eligible for immunomodulating therapy, which may be helpful only in the acquired form (ie, when anti-Ro/SSA are found in the patient).^7^ Therefore, in consideration of the absence of symptoms and of cardiac, laboratory, and genetic abnormalities; no circulating autoantibodies; and the stability of the PR interval, he was deemed eligible for sports competition. We did not recommend additional invasive studies because the patient did not present any tachyarrhythmia or signs of accessory pathways. We advised him to report for yearly follow-up visits, including ambulatory ECG monitoring to exclude the possibility of conduction disturbance progression, because the autoimmune-mediated congenital injury could accelerate the physiological senescence of the conduction system.^7^

During the follow-up period, the athlete has continued to practice competitive sports without events.

## Discussion

The most recognized form of autoimmune AV block is congenital (neonatal lupus), secondary to a passive autoimmunity process in which transplacental passage of maternal anti-Ro/SSA-antibodies mediates structural damage to the conduction system tissues of the fetus.^8^^,^^9^ Growing evidence supports the existence of another presentation for congenital autoimmune AV blocks, with a subclinical presentation in utero/at birth/in the neonatal phase and a manifest conduction disease later in life, in a preschool or an adult phase (late progressive congenital).^8^, ^9^, ^10^ In the case of young athletes with conduction defects not fully explained by training-induced remodeling, it is of paramount importance to exclude secondary forms, including autoimmunity. Therefore, in selected cases, we suggest screening the patient’s and the mother’s serum for anti-Ro/SSA-antibodies, also because currently available tests (immunoenzymatic assays and immunoblotting) are highly sensitive and specific (both up to approximately 95%).^6^ Three scenarios are then possible: circulating antibodies in the patient only (acquired form), in the mother only (late progressive congenital form), or both (mixed form).^5^^,^^7^ The differential diagnosis is clinically relevant, given that acquired forms (and, partially, mixed forms) may be reverted with immunomodulating therapies, whereas congenital late-presenting forms may progress to a greater extent during the years.^7^

This case report describes our investigation of the autoimmunity status of the patient and his mother, supposing that the conduction disturbance was too pronounced to be explained only by sports practice. After structural cardiomyopathies and channelopathies were excluded, the serologic test revealed a scenario compatible with a late-presenting congenital AV block. Therefore, it was possible to identify at least 1 of the probable mechanisms of conduction disturbance, to exclude an acquired autoimmune form possibly responsive to immunomodulating therapies, and to schedule follow-up visits to consider the known potential progression of the block.

## Conclusions

In athletes with marked prolongation of the PR interval, it is of paramount importance to conduct a comprehensive evaluation because training-induced electrical remodeling is rarely the cause of this anomaly. We report in this athlete for the first time, to our knowledge, a late progressive congenital form of autoimmune AV block due to maternal anti-Ro/SSA-autoantibodies, suggesting, in selected cases, the importance of a more comprehensive investigation, also including the assessment of the autoimmunity status of the patient and the mother.

## Funding Support and Author Disclosures

The authors have reported that they have no relationships relevant to the contents of this paper to disclose.
